# Medical News and Miscellany

**Published:** 1897-09

**Authors:** 


### Medical News and Miscellany.

**Dr. E. P. Edwards** has removed from Paris to Bonham.

**Dr. A. E. Morriss** has removed from Burton to Houston.

**R. C. Youngblood** has removed from Wills Point to Noonday, Texas.

**Dr. T. W. Conerly** of San Angelo is in New York City taking a post graduate course.

**Dr. Ed. L. Wedemeyer,** of the class of '97, Medical Department University of Texas, has located at Buckhorn, Texas.

**The Treasury Department** has just issued as a supplementary rule for quarantine officers, that at all stations formaldehyd shall be used as a disinfectant.

**Dr. J. D. Field,** of Manor, Texas, has filed suit for criminal libel in the district court against the publishers of the *Democrat*, a paper recently removed from Manor to Austin. The ground for action is said to have been an abusive and defamatory article which appeared in that paper recently. So says the *Statesman.*

**The Travis County Medical Society**, at a meeting held in Austin Sept. 2, 1897, adopted the following resolution:

*Resolved*, That the Travis County Medical Society favors the appointment, by the Regents, of Texas physicians to fill the existing vacancies in the Medical Department in the University of Texas at Galveston.

---

**Roster of the Medical Officers** of the Texas Volunteer Guard:

The Texas Volunteer Guard consists of one division of two brigades of three regiments of infantry, each, and one regiment of cavalry, and one battalion of artillery. The medical officers are as follows:

Col. R. M. Swearingen, Austin, Surgeon-General.

Lt.-Col. F. C. Ford, Nacogdoches, Medical Director of Division.

Maj. W. A. Lockett, Brenham, Surgeon 1st Brigade.

Maj. W. L. Marshall, Longview, Surgeon 2nd Brigade.

Capt. (vacancy), 1st Regiment.

Capt. I. N. Suttle, Corsicana, Asst. Surgeon 2nd Regiment.

Capt. E. H. Gray, Milano, Asst. Surgeon 3rd Regiment.

Capt. (vacancy), 4th Regiment.

Capt. W. B. McLaughlin, Jefferson, Asst. Surg. 5th Regiment.

Capt. H. T. Herndon,———, Asst. Surgeon 6th Regiment.

Capt. G. W. Larendon, Houston, Asst. Surgeon, 1st Cavalry.

Capt. J. D. Westerveldt, Jr., Dallas, Asst. Surgeon Artillery Battalion.

